# Analysis of gut microbiota of obese individuals with type 2 diabetes and healthy individuals

**DOI:** 10.1371/journal.pone.0226372

**Published:** 2019-12-31

**Authors:** Aftab Ahmad, Wanwei Yang, Guofang Chen, Muhammad Shafiq, Sundus Javed, Syed Shujaat Ali Zaidi, Ramla Shahid, Chao Liu, Habib Bokhari

**Affiliations:** 1 Department of Biosciences, COMSATS University, Chak Shahzad, Islamabad, Pakistan; 2 Endocrine and Diabetes Center, Affiliated Hospital of Integrated Traditional Chinese and Western Medicine, Nanjing University of Chinese Medicine, Jiangsu Province Academy of Traditional Chinese Medicine, Nanjing, China; University of California Los Angeles, UNITED STATES

## Abstract

Type 2 diabetes mellitus (T2DM) accounts for 90% of diabetes cases worldwide. The majority of T2DM patients are obese. Dysbiosis in the gut microflora is strongly associated with the pathogenesis of obesity and T2DM; however, the microbiome of obese-T2DM individuals in the Pakistani population remains unexplored. The gut microbiota signature of 60 Pakistani adults was studied using 16S rRNA sequencing targeting V_3_–V_4_ hypervariable regions. The sequence analysis revealed that bacteria from *Firmicutes* were predominant along with those from *Clostridia* and *Negativicutes*, whereas bacteria from *Verrucomicrobia*, *Bacteroidetes*, *Proteobacteria*, and *Elusimicrobia* were less abundant among the obese T2DM patients. These data distinctively vary from those in reports on the Indian population. The difference in gut microbiota could presumably be related to the distinct lifestyle and eastern dietary habits (high carbohydrate and fat intake, low fiber intake) and unregulated antibiotic consumption. This is the first study carried out to understand the gut microbiome and its correlation with individual life style of obese T2DM patients in the Pakistani population.

## Introduction

The prevalence of diabetes mellitus is increasing at an alarming rate worldwide with more than 422 million people diagnosed with the disease, 90% of which have type 2 diabetes mellitus (T2DM) [1]. Obesity-induced insulin resistance is one of the primary causes of this metabolic disorder [2], with over 90% patients being overweight [3]. In addition to being governed by genetic and environmental factors, T2DM is also influenced by the patient’s gut microbiota specifically, their abundance and diversity [4–6].

The role of gut microbes in the synthesis of essential vitamins, amino acids, and degradation of toxins has been studied [7]. However, during the past decade, evidence has suggested that the gut microbiome plays a crucial role in diseases and dysbiosis and is significantly associated with T2DM [3]. Interestingly, large-scale 16S rRNA sequencing studies revealed a strong association between the gut microbiota composition and metabolic disorders such as obesity and T2DM [8,9]. More than 1000 species of bacteria belonging to different phyla reside in the human gut, the dominant ones being *Firmicutes* and *Bacteroidetes* together with *Actinobacteria* and *Proteobacteria* [10,11]. The aforementioned phyla enhance the uptake of monosaccharides, thus elevating the production of hepatic triglycerides, which in turn trigger insulin resistance [12]. Additionally, *Bacteroides* have strong positive correlation with intake of fat-rich diet [13–15]. The disturbance in relative bacterial abundance and diversity eventually results in metabolic disorders such as inflammatory bowel disease, diabetes mellitus, Crohn’s disease, and multiple sclerosis [16]. Previous studies have also reported that the gut microbiome has a strong association with T2DM [6,7,10]. The gut microbiota of T2DM patients was characterized by reduced diversity with increased proportion of *Firmicutes* relative to *Bacteroidetes*, in reports from the populations of eastern countries (e.g., Japan and Saudi Arabia) [17–19] compared to that in the Western population where this trend was not consistently observed in different studies [8,20–22].

The dysbiosis may lead to metabolic disorders including T2DM by hampering the production of carbohydrate metabolism end products specifically associated with decreased number of butyrate-producing microbes [23]. Acetate and butyrate are short chain fatty acids (SCFAs); butyrate can modulate immune response and cause low-grade inflammation and could be one of the causes of T2DM. Therefore, differences in the gut microbiota could be influenced by ethnic, environmental, dietary, and socioeconomic factors. The majority of the aforementioned factors can vary greatly among different ethnic groups worldwide [24]. Furthermore, gut microbial communities (*Clostridium hystoliticum*, *Eubacterium rectale*, and *Clostridium coccoides*) can affect neurotransmitters involved in gut–brain signaling pathways, and thus, regulate food intake and body weight. These neurotransmitters directly control the intestinal transit time, serotonin levels, physical activity, and intestinal permeability [4,25,26].

There is a paucity of information available on gut microbiota composition of Muslim populations with low socioeconomic status and consumption of a typical eastern diet. In a preliminary study in 2018, Batool et al. examined the gut microbiome structure of healthy individuals in the Pakistani population [27]. The present study is the first to report the gut microbiota of obese Pakistani individuals with T2DM.

## Material and methods

### Sample collection

Blood samples were collected during the first quarter of 2017 from the diabetic clinic of the Social Security Hospital, Islamabad. All volunteers were interviewed and their age, gender, weight, ethnicity, diet, medical/drug history, fasting glucose level, lipid profile, smoking, physical activities, and alcohol intake was documented. Fasting blood glucose and lipid profile (triglycerides, total cholesterol, HDL, and LDL) were measured using an automated enzymatic analyzer (Cobas Integra 700; Hoffman-La Roche, Basel, Switzerland).

In general, adults aged 25–55 years, belonging to ethnic Punjabi (inhabitants of largest province) population of Pakistan, were included in the present study. Participants suffering from any symptoms of constipation, bloody stool, diarrhea, or any other gastrointestinal disease and those who were administered antibiotics (oral or injectable) in past three months, were excluded. All stool samples were collected within 24 h after compiling the questionnaire. Samples were collected in sterile containers provided to the volunteers and were stored at −80 °C. Sampling was performed using all standard protocols and regulation.

### Data collection, 16S rRNA sequencing, and quality control

Microbial DNA was isolated from 200 mg of each homogenized fecal sample, following the protocols of TIAGEN^®^ DNA Stool kit (Tiagen, China). It was then quantified using Nanodrop spectrophotometer (Nanodrop Technologies, USA). Isolated DNA was stored at −20 °C for further study. The V3–V4 region of 16S rRNA gene was amplified in each sample using universal primers 343 F (5′-TACGGRAGGCAGCAG-3′) and 798 R (5′-AGGGTATCTAATCCT-3′) [28]. The resulting amplicons (456 bp) were subsequently subjected to high-throughput sequencing using the Illumina MiSeq platform. All low quality (<Q20) terminal bases were removed using Trimmomatic (v0.35), thus retaining the high quality reads only. All sequencing results after quality control, which are used in the project along with the metadata, have been submitted to Sequence Read Archive (SRA) NCBI database (accession number PRJNA554535).

### Analysis of bacterial composition

The filtered high-quality reads were stitched by using FLASH (v1.2.11). Script multiple_join_paired_ends.py of QIIME (1.8.0) was used to assemble the 16S rRNA amplicons. Moreover, mismatched barcodes and sequences with length less than the threshold (200 bases), were removed. Chimeric sequences were removed using UCHIME (v4.2) providing valid tags (a high-quality sequence) for downstream microbial diversity analysis. These quality-filtered reads were clustered into operational taxonomic units (OTUs; 97% identity) using *de novo* OTU picking and taxonomic assignment by implementing VSEARCH (v2.4.2) against Silva (v123), and Greengenes (gg13.8) reference databases utilizing QIIME script (pick_de_novo_otus.py). The representative sequences were classified and annotated by the Naive Bayesian classification algorithm of RDP classifier (v2.2).

### Diversity index calculation statistics

**Fig 1:**
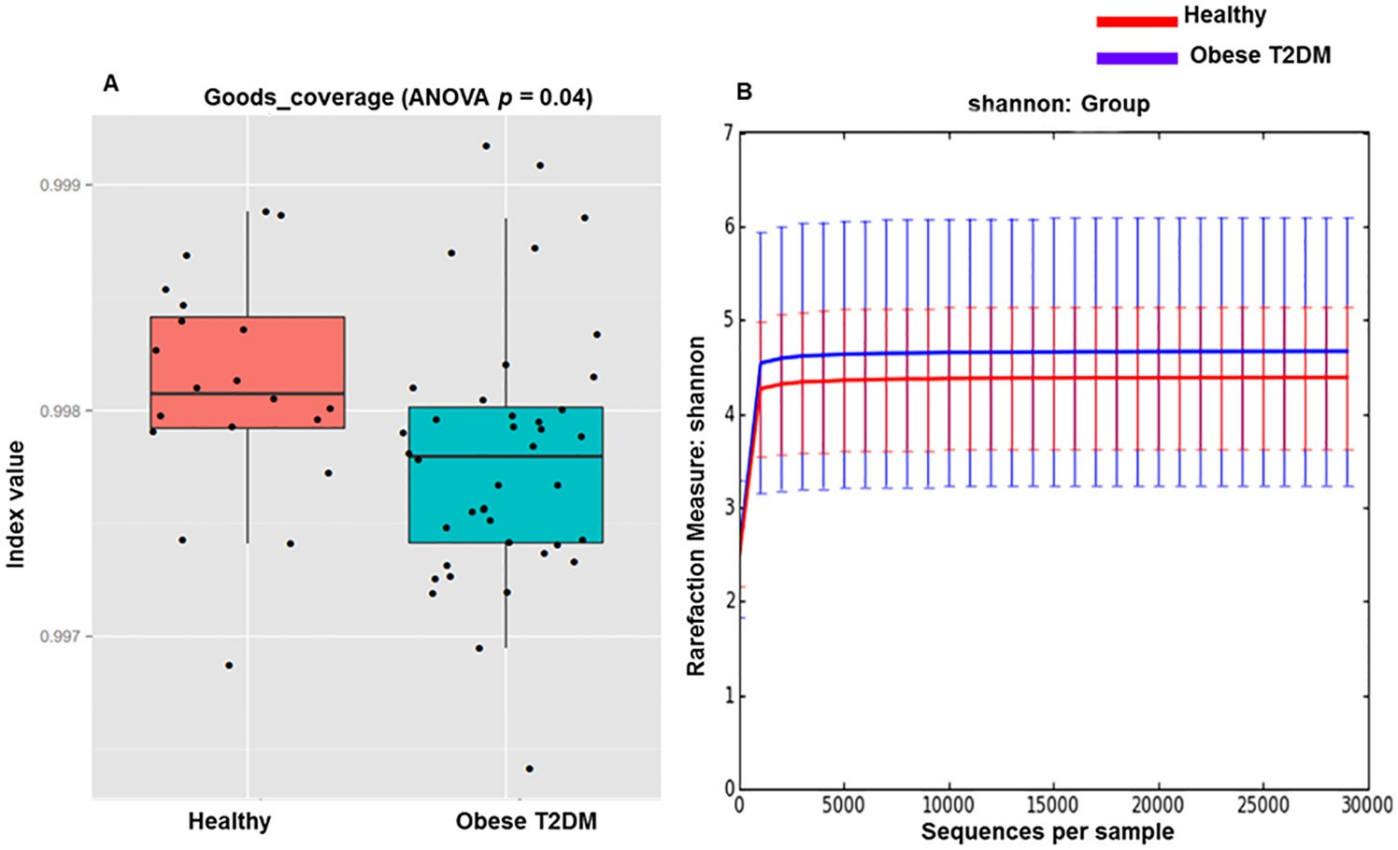
Alpha diversity between obese-T2DM patients. (A) Comparison of Boxplots depicting OTU/Diversity between obese-T2DM patients (*n* = 40) and healthy participants (*n* = 20). (B) The Shannon Wiener index representing the number of species and uniformity of individual distribution between obese-T2DM patients (*n* = 40) and healthy participants (*n* = 20).

QIIME was used to perform diversity analysis, using the default parameters. Filtration and closed-reference OTU clustering ended with a variable count, possessing a wide range of sequences per sample. To address this randomness, the sequence data was rarefied to 829 OTUs per sample for alpha and beta diversity analyses. Furthermore, the diversity boxplot diagram and ANOVA was used to find the diversity and Shannon index in order to enumerate the species count in obese-T2DM and healthy individuals (Fig 1). Additionally, species accumulation curve was used to determine the sampling depth (S1A Fig), and the sample diversity and degree of uniformity were measured via rank abundance curve method (S1B Fig).

### α-diversity and β-diversity analysis

**Fig 2:**
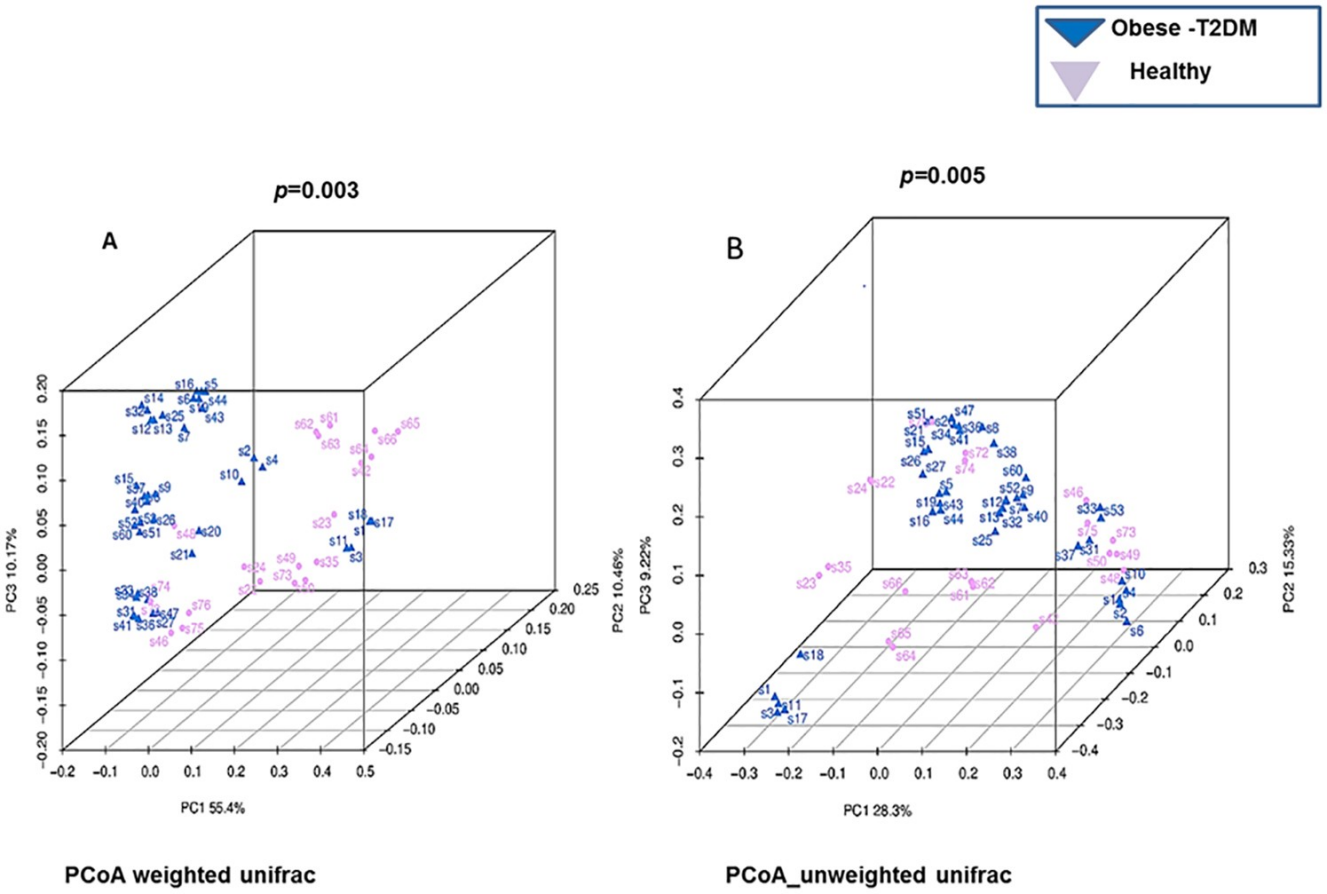
Beta-diversity of the gut microbial communities in obese-T2DM patients and healthy participants. Principal Coordinates Analysis (PCoA) plot based on weighted and unweighted UniFrac distance. Each dot represents one sample from each group.

α-Diversity (diversity within samples) was analyzed based on rarefied data (using minimum number of sequences among samples), using the PD whole-tree box plot, observed species box plot and Chao1 (frequency-based richness estimator) (S2A–S2C Fig). β-diversity (diversity between samples) was assessed by weighted UniFrac, unweighted UniFrac, and Bray Curtis distances. Adonis analysis was used to evaluate the significance between groups (S1 Table). A Principal Coordinate Analysis (PCoA) plot, based on weighted-UniFrac and unweighted-UniFrac and Bray-Curtis distance matrices was constructed to demonstrate the overall dissimilarity of bacterial communities in the two groups of individuals from the Pakistani population (Fig 2).

### Statistical analysis

Welch *t-test* was used to calculate significant differences in age, BMI, and lipid profile of obese-T2DM and healthy individuals (Table 1). Moreover, Kruskal–Wallis rank-sum test was used to compare the relative abundance between obese-T2DM patients and healthy individuals, and false discovery rate (FDR) using the Benjamini-Hochberg method was applied to correct the significant *p*-values (Table 2).

**Table 1:** Anthropometric and biochemical parameters of participants.

Parameters	Obese-T2DM	Healthy	Welch t-test p-value
N = 60	40	20	-
Age (years)	38.1± 12.1	37.7 ± 12.1	> 0.06
BMI (Kg/ m^2^)	32.4 ± 3.6*	22.08 ± 3.1*	< 2.2E-16
Glucose (mg/dL)	221.5 ± 61.5*	84.8± 30.4*	< 2.2E-16
Total cholesterol (mg/dL)	202.2 ± 25.2*	124.3 ± 31.6*	< 2.2E-16
Triglycerides (mg/dL)	164.9 ± 26.4*	85.7± 26.4*	< 2.2E-16
HDL(mg/dL)	46.2 ± 10.4*	36.9 ± 8.5*	= 4.326E-05
LDL(mg/dL)	131.2 ± 33.4*	49.2 ± 27.7*	< 2.2E-16

Results of Welch two sample t-test (obese-T2DM, healthy age, BMI, glucose, cholesterol, triglycerides, HDL, LDL, mu = 0, alt = “two. sided.”, conf. level = 0.95)

* Indicates *p* < 0.05

**Table 2:** The relative abundance of gut microbiota at phylum, class and genus level between obese-T2DM patients (*n* = 40) and healthy participants (*n* = 20) after FDR adjustment using Kruskal-Wallis rank-sum tests.

Bacterial Taxonomy		Mean relative Abundance			*p*-Value	
		ObeseT2DM	Healthy	Test-Statistics	*p*-value	FDR
***Verrucomicrobia***	**Phylum**	**0.0%**	**0.1%**	**11.90**	**0.00002**	**0.0003**
*Verrucomicrobiae*	Class	0.0%	0.1%	18.03	0.00002	0.0006
***Firmicutes***	**Phylum**	**55.7%**	**36.9%**	**11.90**	**0.0005**	**0.004**
*Bacilli*	Class	5.7%	6.5%	4.61	0.03	0.1
*Bacillus*	Genus	0.008%	0.04%	6.30	0.01	0.06
*Lactobacillus*	Genus	2.9%	1.4%	6.29	0.01	0.06
*Clostridia*	Class	36.2%	22.5%	10.84	0.0009	0.01
*Eubacterium coprostanoligenes group*	Genus	7.3%	1.9%	13.23	0.0002	0.006
*Subdoligranulum*	Genus	5.3%	4.1%	5.60	0.01	0.07
*Christensenellaceae_R_7_group*	Genus	1.2%	0.5%	4.38	0.03	0.1
*Ruminococcus_2*	Genus	0.09%	0.1%	9.02	0.002	0.02
*Negativicutes*	Class	9.6%	2.2%	13.00	0.0003	0.004
*Dialister*	Genus	8.8%	1.7%	12.44	0.0004	0.007
*Allisonella*	Genus	0.1%	0.002%	28.20	1.09e07	0.00002
***Bacteroidetes***	**Phylum**	**19.5%**	**32.1%**	**9.73**	**0.001**	**0.009**
*Bacteroidia*	Class	19.5%	32.1%	9.73	0.001	0.01
*Prevotella_9*	Genus	15.4%	23.0%	12.89	0.0003	0.006
***Proteobacteria***	**Phylum**	**8.9%**	**15.8%**	**9.06**	**0.002**	**0.01**
*Gamaproteobacteria*	Class	8.6%	15.0%	9.15	0.002	0.01
*Escherichia_Shigella*	Genus	7.0%	11.9%	6.53	0.01	0.05
*Deltaproteobacteria*	Class	0.02%	0.2%	5.83	0.01	0.06
***Elusimicrobia***	**Phylum**	0.0%	0.00001%	6.20	0.01	0.04
*Elusimicrobia*	Class	0.0%	0.001%	6.20	0.01	0.05
*Coriobacteriia*	Class	8.9%	5.3%	7.53	0.006	0.03

### Ethical approval and participants’ consent

Our analysis was conducted on total 60 fecal samples subdivided as obese-T2DM (40 samples) and lean, non-T2DM, and healthy (20 samples), isolated from Punjabi individuals in Pakistan. Written consent was obtained from all the participants enrolled in the study. The study is approved by the Ethical Research Review Committees of COMSATS University, Islamabad, Pakistan. The experiments were performed according to the standard procedures and regulations.

## Results

### Sequencing output and data preprocessing

The process of filtering and adapter trimming resulted in 2,402,668 high-quality paired-end reads (median read length of 428 bp per sample).

### Anthropometric and biochemical parameters of participants

The age and sex distribution were similar for the obese-T2DM (mean age 38.1± 12.1 years) and healthy individuals (mean age 37.7 ± 12.1 years). The BMI, and fasting blood sugar, total cholesterol, HDL, LDL, and triglyceride levels of obese-T2DM patients were significantly higher as compared to those of the healthy participants (Table 1).

### Association of α-diversity with obese-T2DM status

Alpha diversity was quantified using the Shannon diversity index, which relates both OTU richness and evenness, and by the total number of observed species. Fig 1 depicts the alpha diversity measurements for obese T2DM patients versus controls. Statistical testing presented no difference for the PD_whole tree (pPD_whole = 0.91; S2B Fig), observed species (pObserved = 0.23; S2B Fig), and Chao1 richness estimator (pChao1 = 0.15; S2C Fig), while the Good’s coverage (diversity index) that reflects that the depth of sequencing was significantly decreased in obese-T2DM patients compared to that in the controls (pGoods = 0.04).

### β-diversity of obese-T2DM patients and healthy individuals in Pakistani population

**Fig 3:**
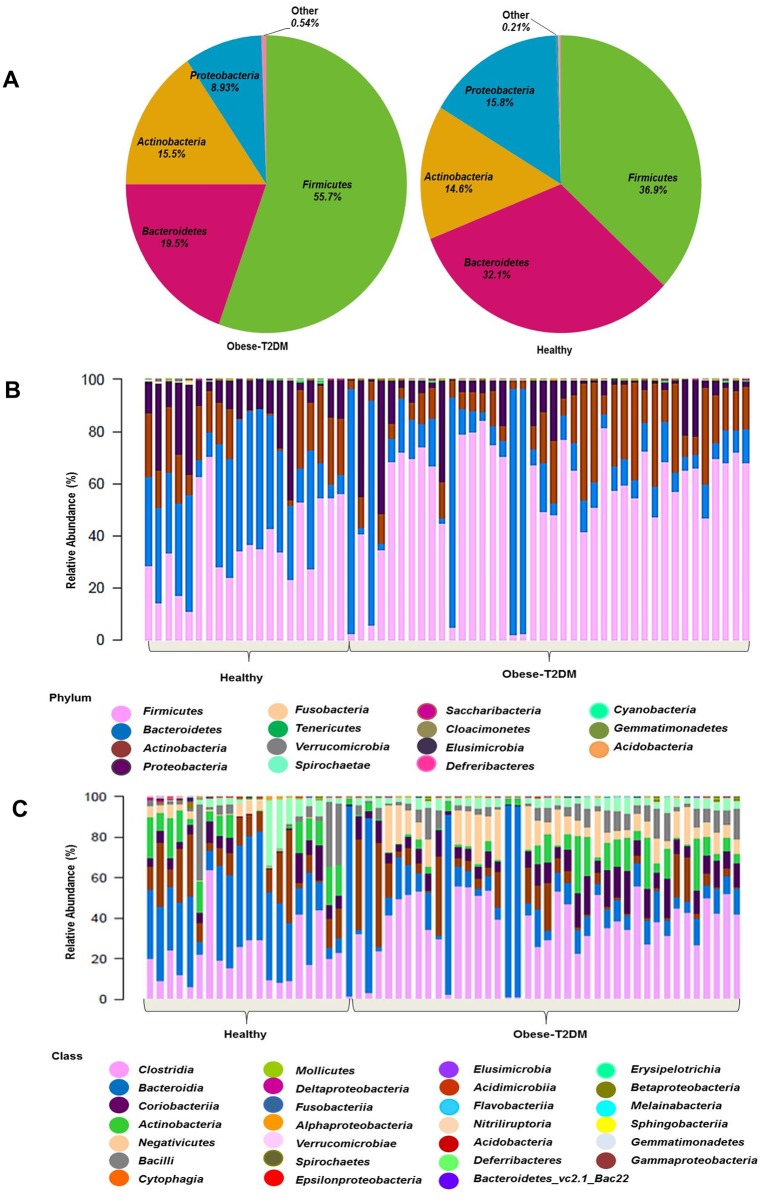
The relative abundance of gut bacteria in obese-T2DM patients and healthy participants using Kruskal-Wallis rank-sum tests. (A) Relative percentage of most abundant phyla between obese-T2DM patients (*n* = 40) and healthy individuals (*n* = 20). (B) Relative percentage of most abundant phyla in each sample between obese-T2DM patients (*n* = 40) and healthy individuals. (C) Relative abundance of bacteria at class level in obese-T2DM patients (*n* = 40) and healthy participants (*n* = 20).

In majority of the obese-T2DM samples, the *Firmicutes* phyla constituted the highest proportion of the bacterial population compared to that in the healthy individuals, with relative abundance of 55.7% and 36.9%, respectively (Fig 3A–3C). Moreover, the relative abundance of *Verrucomicrobia*, *Bacteroidetes*, *Proteobacteria*, and *Elusimicrobia* was less or even completely absent in obese-T2DM patients (Table 2).

Relative abundance of *Clostridia*, *Negativicutes*, and *Coriobacteriia* was higher in obese-T2DM patients. At the genus level, the *Eubacterium coprostanoligenes* group, *Dialister*, and *Allisonella* presented increased relative abundance in obese-T2DM individuals (Table 2).

### Microbial configuration in T2DM presents differential clustering

Microbial community distribution between obese-T2DM and healthy individuals was assessed by Adonis analysis based on permutation pMANOVA to analyze the variance. The weighted UniFrac patterns indicated that the obese-T2DM and healthy individuals clustered separately, thus presenting 55.4% and 10.17% of the total variance on the x-axis and y-axis, respectively. In contrast, the unweighted UniFrac clustering pattern reflects 28.3% and 9.22% of the total variance along the x-axis and y-axis, respectively (Fig 2), and UPGMA tree analysis presented a noticeable difference in the gut microbial compositions between obese-T2DM and healthy groups (S3 Fig).

### Distinct gut microbiota in obese-T2DM individuals

As indicated in the Shannon index (Fig 1), a non-significant correlation of the microbial community richness exists between obese-T2DM and healthy individuals. An evident difference in the gut microbiota, at all taxonomic levels, was observed between these two groups (Table 3). The presence of *Verrucomicrobia* and *Elusimicrobia* exclusively, in the healthy Pakistani population revealed positive correlation with the healthy controls (Table 3). Similarly, the exclusive presence of *Acidobacteria*, *Deferribacteres*, *and Gemmatimonadetes* in the obese-T2DM individuals demonstrated their positive association with the diabetes state (Table 3). Furthermore, numerous bacterial species namely, *Prevotella* P4_76, *Clostridiales bacterium canine oral taxon* 100, *Porphyromonadaceae bacterium DJF B175*, *rumen bacterium RF9*, *Candidatus Alistipes marseilloanorexic AP11*, *bacterium ASC802*, *Bacillus sporothermodurans*, *Staphylococcus SV3*, and *Iamia*, were present in the obese-T2DM group of individuals only; whereas, *methanogenic archaeon* was absent in the diseased individuals and was observed only in healthy individuals (Table 3).

**Table 3:** Gut microbiota present exclusively in either obese-T2DM patients (*n* = 40) or healthy participants (*n* = 20).

Bacterial Taxonomy	Annotation	Obese-T2DM	Healthy	Test- statistics	*p*-value	FDR
*Verrucomicrobia*	Phylum	0	0.001	18.03	0.00002	0.0003
*Elusimicrobia*	Phylum	0	0.00001	6.205	0.01	0.04
*Acidobacteria*	Phylum	2.93E-06	0	0.5	0.4	0.6
*Deferribacteres*	Phylum	2.26E-06	0	0.5	0.4	0.6
*Gemmatimonadetes*	Phylum	1.61E-06	0	0.5	0.4	0.6
*Prevotella_sp*.*_P4_76*	Species	0.000136	0	8.00	0.004	0.05
*uncultured_methanogenic_archaeon*	species	0	0.00005	6.20	0.01	0.08
*Clostridiales_bacterium_canine_oral_taxon_100*	species	8.49E-06	0	4.50	0.03	0.1
*Porphyromonadaceae_bacterium_DJF_B175*	species	1.19E-05	0	3.87	0.04	0.1
*unidentified_rumen_bacterium_RF9*	species	0.000349	0	3.26	0.07	0.1
*bacterium_ASC802*	species	1.51E-06	0	1.01	0.3	0.4
*Candidatus_Alistipes_marseilloanorexicus_AP11*	species	6.00E-06	0	1.55	0.2	0.3
*Bacillus_sporothermodurans*	species	1.53E-06	0	0.5	0.4	0.6
*Staphylococcus_sp*.*_SV3*	species	1.61E-06	0	0.5	0.4	0.6
*uncultured_Iamia_sp*.	species	2.27E-06	0	0.5	0.4	0.6

### Correlations between gut microbiota in fecal samples and fasting glucose levels

**Fig 4:**
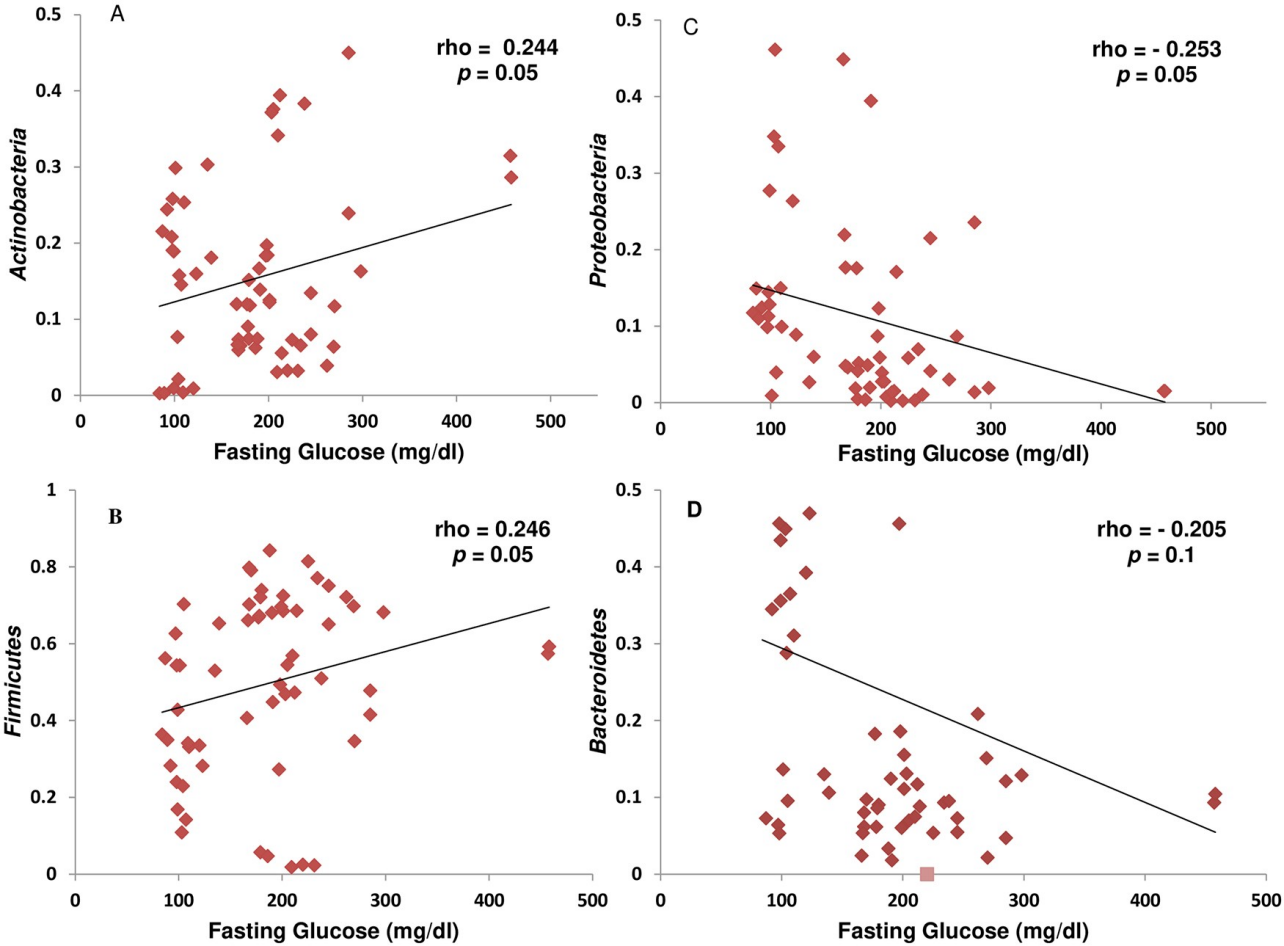
Correlations between fasting glucose and gut microbiota in fecal samples of participants. Spearman’s rank correlation coefficient was used to analyze the correlation between obese-T2DM (*n* = 40) and healthy (*n* = 20). (A) Correlation between fasting glucose and phylum *Actinobacteria*. (B) Correlation between fasting glucose and phylum *Firmicutes* (ρ = 0.246, p = 0.05). (C) Correlation between fasting glucose and phylum *Proteobacteria* (ρ = -0.253, p = 0.05). (D) Correlation between fasting glucose and *Bacteroidetes* (ρ = -0.205, p = 0.11).

Spearman’s rank correlation is a univariate analysis that was carried out with adjusted weight to evaluate the possible effect of glucose metabolism on gut microbial abundances (Fig 4). It was revealed that the relative abundance of *Actinobacteria* and *Firmicutes* was positively correlated with fasting glucose levels (Fig 4A and 4B), whereas that of *Proteobacteria* and *Bacteroidetes* demonstrated a negative correlation (Fig 4C and 4D).

## Discussion

This is the first study to characterize the gut microbiome of obese-T2DM and healthy individuals in the Pakistani population. A total of 60 participants (40 obese-T2DM and 20 healthy) were recruited in the study, from January to March 2017. We evaluated the diversity, richness, and compositional variation of the gut microbiome among the healthy controls and obese-T2DM groups from the Punjabi Pakistani population. In contrast to those among the healthy individuals, the baseline clinical characteristics such as fasting glucose concentration, total cholesterol, triglycerides, HDL, LDL, and BMI were significantly high among individuals with obese-T2DM. Elevated levels of HDL and triglycerides are the most common factors associated with metabolic disorders in 85.5% of the Pakistani obese and T2DM individuals [29]. This is presumably due to frequent intake of eastern food comprising carbohydrate-rich compounds and edible oils, which are an essential part of the Pakistani diet [27].

We observed an altered microbial composition in obese-T2DM individuals, thus suggesting a distinct signature of microbiota associated with T2DM in obese individuals from the Pakistani population. Previous studies on healthy individuals in the Pakistani population reported that the microbiome of Pakistani individuals is more similar to that of people from Western countries as compared to that of the Indian population [27,30]. Moreover, the microbiomes of obese-T2DM individuals in the Pakistani population revealed an altered composition with an increased abundance of *Firmicutes* (55.7%), whereas *Proteobacteria* (58.8%) is reported to dominate the gut microbial diversity of obese-T2DM individuals from India [31]. Furthermore, at the generic level, *Prevotella*_9 and *Eubacterium coprostanoligenes* demonstrated a declining trend in the obese-T2DM Pakistani individuals compared to those from India [31]. This study sets a benchmark for the comparative analysis of obese-T2DM and healthy metagenomic samples in the Pakistani population.

We discovered a high percentage of *Firmicutes* and a reduced abundance of *Bacteroidetes* in the obese-T2DM samples similar to that in the earlier studies [8,9,12,32–34]. Furthermore, *Firmicutes* and *Actinobacteria* demonstrated a positive correlation with the fasting glucose levels, whereas the abundance of *Bacteroidetes* and *Proteobacteria* presented a negative relationship [35]. The *Firmicutes* are well known for fat digestion, and their increased abundance is also known to be associated with obesity. Similarly, *Bacteroidetes* play a crucial role in producing short-chain fatty acids (SCFAs), and *Actinobacteria* execute a key role in the biodegradation of resistant starch. It is also suggested that *Firmicutes* and *Bacteroidetes* enhance the monosaccharide uptake from the host gut, which elevates the production level of hepatic triglycerides, thereby resulting in insulin resistance [9,20,33]. This change may contribute positively to low-grade inflammation mainly achieved via activation of SCFA-linked G-protein-coupled receptors (GPCR), thus leading to metabolic disorders [12,36,37]. Additionally, resistance to resistin due to prolonged gut dysbiosis makes an individual susceptible to insulin resistance [38]. Factors such as ethnicity or genetic makeup may contribute to an increased susceptibility of Pakistani individuals to insulin resistance. In a comparative study between Pakistani immigrants and Norwegian individuals with T2DM in Norway, despite the fact that Pakistani immigrants had lower BMI and body fat mass (BFM) than Norwegians, they were more susceptible to insulin resistance and inflammation [39].

Although numerous bacterial species were particularly present or absent in the obese-T2DM samples, due to low sequencing depth and few number of samples, we could not effectively compare the species-level composition. Nevertheless, our preliminary findings at the species level provide interesting evidence warranting further investigations. For instance, *Prevotella* P4 76 was the only bacterial species observed particularly in the obese-T2DM individuals. The species is involved in elevating levels of proinflammatory cytokines, onset of low-grade inflammation, and insulin resistance [40–43].

Another important observation was the absence of *Verrucomicrobia* from obese-T2DM samples, and this phyla is considered as an efficient agent in maintaining the anti-inflammatory state of gut and improved insulin sensitivity [44]. Moreover, *Verrucomicrobia* is known to improve glucose metabolism in animals [45]. The depletion of *Verrucomicrobia* might have resulted in growth of rogue *Protobacteria*, such as *Escherichia and Shigella*. This notable change might contribute toward the development of T2DM [12].

The comparison of obese-T2DM and healthy microbiome samples during the present study suggested significantly reduced abundance of *Gammaproteobacteria* in obese-T2DM. Additionally, we observed that the abundance of gram-negative bacteria (*Dialister and Allisonella*) increased drastically in the obese-T2DM samples from the Pakistani population. These bacteria are extremely important for their contribution toward the development of disease as their increased abundance might have elevated the lipopolysaccharide (LPS) level in obese-T2DM individual. The circulating LPS binds with CD14 and mediates an inflammatory response, thus contributing positively to the development of obesity and insulin resistance [46–49]. We also detected an abundance of class *Fusobacteria* in the obese-T2DM samples. This particular class plays a pivotal role in energy generation, mounting adhesiveness to host epithelial cells, and inflammatory responses [50,51]. Studies suggest that high abundance of *Fusobacteria* is contributing significantly in T2DM along with other conditions [52].

The recruitment of healthy individuals from the Pakistani population was difficult as >85% of the individuals interviewed did not fall in the normal BMI range. This was due to several factors including physical inactivity, poverty (malnutrition), dietary habits, and overeating. A recent survey also revealed that around 60% of the Pakistani population are inactive [53]. Frequent unregulated use of antibiotics was another reason for the exclusion of several healthy volunteers from the present study.

Although, this is a preliminary study to explore the obese-T2DM gut microbiome in comparison to the healthy gut microbiome of Pakistani population, it includes certain limitation such as low sample number as well as sequencing depth. The majority of the participants were sampled only once, a time-series monitoring of multiple samples at various time points and of a higher number of participants would provide more insights that might have been missed due to low sequencing depth.

## Conclusion

This preliminary study based on a small sample size depicts the obese-T2DM microbiome in the Pakistani population. Our study revealed high variability in the microbiome of healthy and obese-T2DM individuals within this population. The variation between normal and obese-T2DM fecal microbiome trends have been reported in other studies as well. Nonetheless, the significance of the present study cannot be undervalued as it provides the scientific community with a first glimpse of the signature microbiota associated with Pakistani obese-T2DM individuals. Furthermore, it also provides the road map for future studies. We believe that this signature of obese-T2DM individuals in the Pakistani population will aid future studies.

## Supporting information

S1 Fig **(A) Alpha Diversity: Species Accumulation Curve for each sample**, the horizontal axis represents the sample size, and the vertical axis represents the number of OTUs detected in the sample. Cross symbols represent the possibility of new species (OTU) addition, with the addition of new samples. (B) Rank Abundance curve was used to explain sample diversity and abundance of species in each sample. The abundance of species is reflected by the length of the curve on the horizontal axis while uniformity of species composition is reflected by the shape of the curve.
(TIF)

S2 Fig Alpha diversity box plots of intestinal microbiota within obese-T2DM patients and healthy (n = 20) participants. (A) PD_whole_tree measures species richness or diversity (B) Observed Species indicates the number of actually observed species (C) Chao1 estimates observed species frequency.
(TIF)

S3 Fig Unweighted Pair Group Method with Arithmetic Mean (UPGMA) method representing beta diversity between obese-T2DM and healthy groups. (TIF)

S1 Table Adonis unweighted and weighted Unifrac results, based on the distance matrix algorithm. R2: indicates the degree of interpretation of the difference between the different groups, that is, the ratio of the variance of the group to the total variance.
(DOCX)
