# Supplementary figures and images for: Analysis of gut microbiota of obese individuals with type 2 diabetes and healthy individuals
Source: PLoS One. 2019 Dec 31;14(12):e0226372. doi: 10.1371/journal.pone.0226372 (PMC6938335; doi:10.1371/journal.pone.0226372)

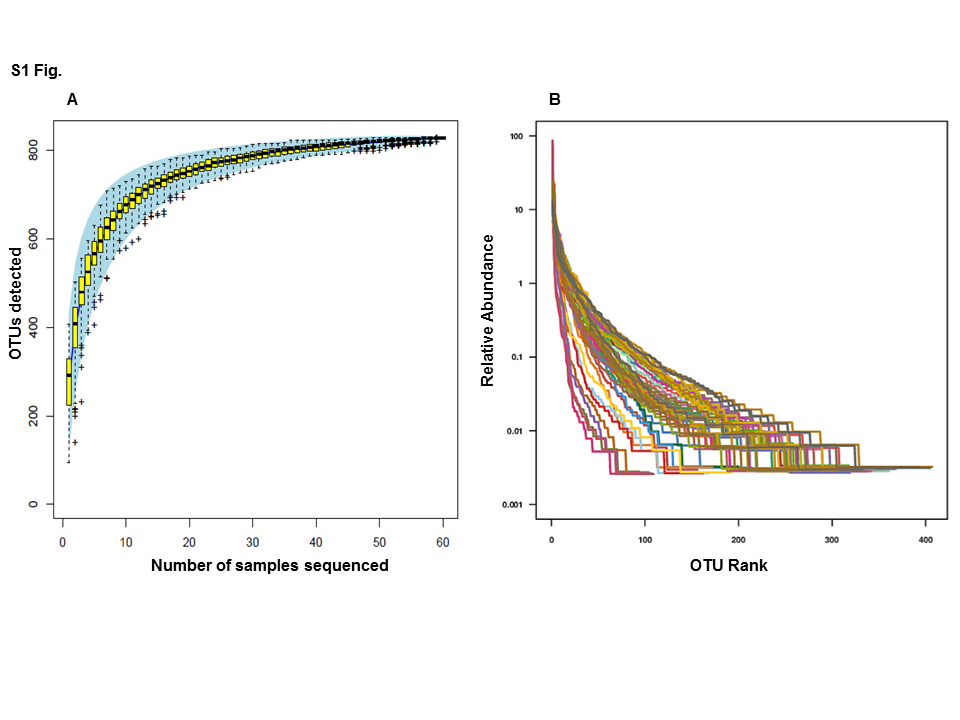

Supplement: S1 Fig — (A) Alpha Diversity: Species Accumulation Curve for each sample, the horizontal axis represents the sample size, and the vertical axis represents the number of OTUs detected in the sample. Cross symbols represent the possibility of new species (OTU) addition, with the addition of new samples. (B) Rank Abundance curve was used to explain sample diversity and abundance of species in each sample. The abundance of species is reflected by the length of the curve on the horizontal axis while uniformity of species composition is reflected by the shape of the curve. (TIF) [file pone.0226372.s001.tif]

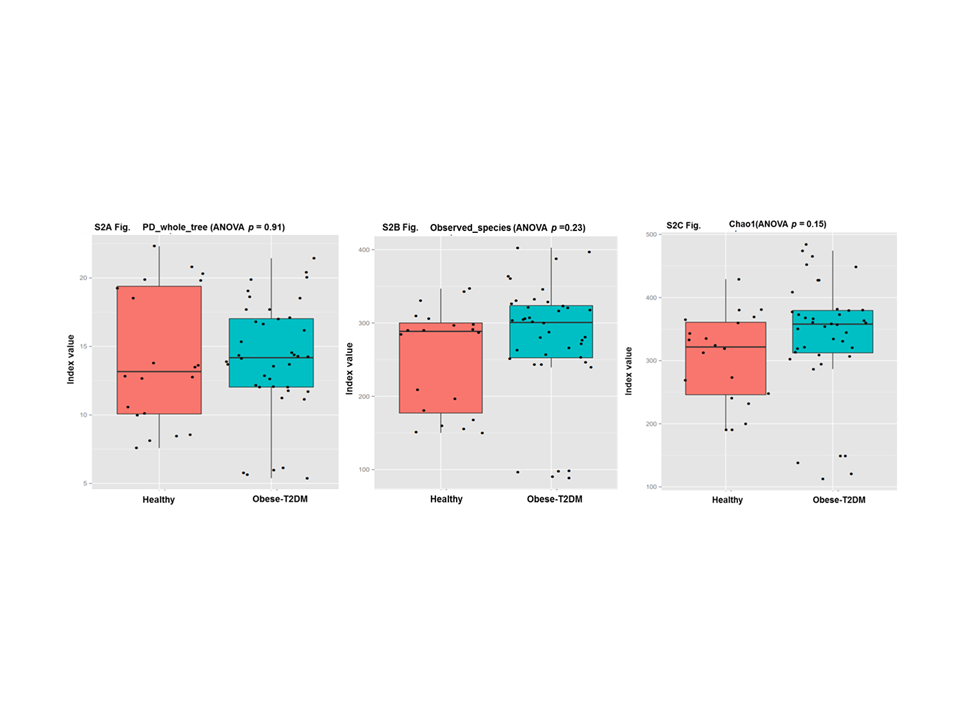

Supplement: S2 Fig — (A) PD_whole_tree measures species richness or diversity (B) Observed Species indicates the number of actually observed species (C) Chao1 estimates observed species frequency. (TIF) [file pone.0226372.s002.tif]

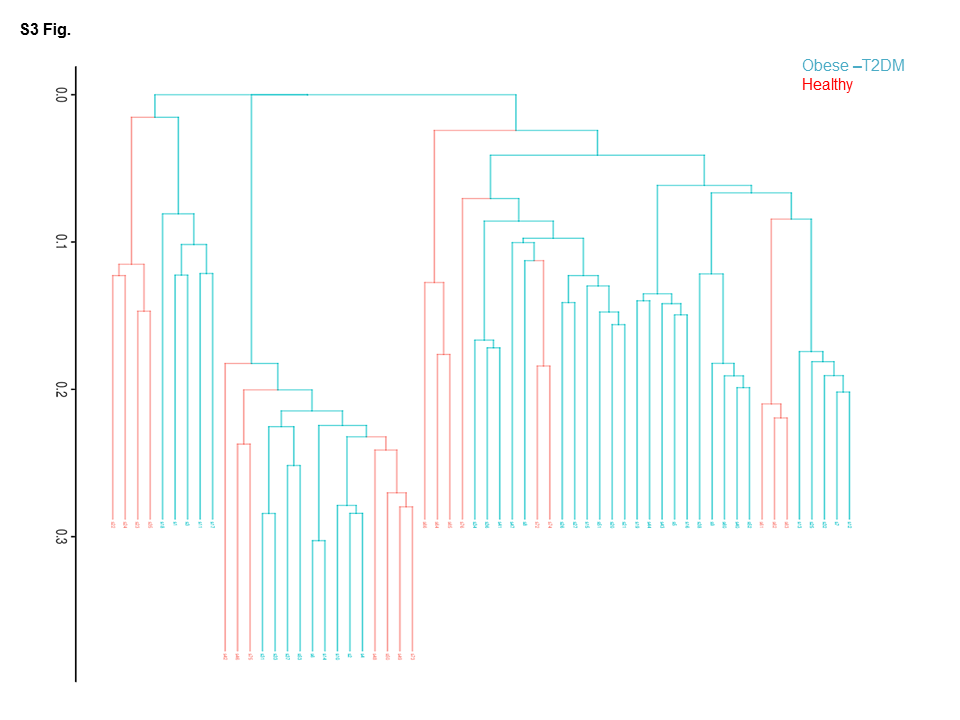

Supplement: S3 Fig — (TIF) [file pone.0226372.s003.tif]
