# Supplementary material for: Analysis of gut microbiota of obese individuals with type 2 diabetes and healthy individuals
Source: PLoS One. 2019 Dec 31;14(12):e0226372. doi: 10.1371/journal.pone.0226372 (PMC6938335; doi:10.1371/journal.pone.0226372)
Supplement: S1 Table — R2: indicates the degree of interpretation of the difference between the different groups, that is, the ratio of the variance of the group to the total variance. (DOCX) [file pone.0226372.s004.docx]

**S1 Table. Adonis Unweighted and weighted Unifrac results**

| **Unweighted** | **Df** | **SumsOfSqs** | **MeanSqs** | **F.Model** | **R2** | **Pr(>F)** | **Significance** |
| --- | --- | --- | --- | --- | --- | --- | --- |
| Group Factor | 1 | 0.3979 | 0.3979 | 2.8005 | 0.04606 | 0.005 | ** |
| Residuals | 58 | 8.2409 | 0.14208 |  | 0.95394 |  |  |
| Total | 59 | 8.6388 |  |  | 1.0000 |  |  |
| **Weighted** |  |  |  |  |  |  |  |
| Group Factor | 1 | 0.35094 | 0.35094 | 7.2712 | 0.1114 | 0.003 | ** |
| Residuals | 58 | 2.79933 | 0.04826 |  | 0.8886 |  |  |
| Total | 59 | 3.15027 |  |  | 1.0000 |  |  |
| **Bray_Curtis** |  |  |  |  |  |  |  |
| Group Factor | 1 | 1.4014 | 1.40143 | 6.0358 | 0.09426 | 0.001 | *** |
| Residuals | 58 | 13.4669 | 0.23219 |  | 0.90574 |  |  |
| Total | 59 | 14.8683 |  |  | 1.0000 |  |  |

**Description of the header**: [1] Df: indicates degree of freedom; [2] SumsOfSqs: indicates total variance, also known as squared deviation; [3] MeanSqs: indicates mean square (poor), i.e. SumsOfSqs/Df; [4] F .Model: indicates the F test value; [5] The larger the R2, the higher the degree of interpretation of the difference between the groups; [6] Pr: Indicates the *p*-value, which is less than 0.05, indicating that the testability of this test is high, indicating that the level of the group is statistically significant; [7] Significant mark, 0.001- 0.01 is marked as **
